# Crystal structure of 2,2-dimethyl-*N*-(5-methyl­pyridin-2-yl)propanamide

**DOI:** 10.1107/S2056989015009378

**Published:** 2015-05-23

**Authors:** Gamal A. El-Hiti, Keith Smith, Amany S. Hegazy, Saud A. Alanazi, Benson M. Kariuki

**Affiliations:** a Cornea Research Chair, Department of Optometry, College of Applied Medical Sciences, King Saud University, PO Box 10219, Riyadh 11433, Saudi Arabia; b School of Chemistry, Cardiff University, Main Building, Park Place, Cardiff CF10 3AT, Wales

**Keywords:** crystal structure, propanamide, hydrogen bonding

## Abstract

There are two mol­ecules in the asymmetric unit of the title compound, C_11_H_16_N_2_O. The pyridine rings and amide groups overlap almost perfectly (r.m.s. overlay fit = 0.053 Å), but the tertiary butyl groups have different orientations: in one mol­ecule, one of the methyl C atoms is *syn* to the amide O atom [O—C—C—C = −0.8 (3)°] and in the other the equivalent torsion angle is 31.0 (2)°. In the crystal, the two independent mol­ecules are linked by a pair of N—H⋯N hydrogen bonds in the form of an *R*_2_^2^(8) loop to form a dimer. A C—H⋯O inter­action connects the dimers into [100] chains.

## Related literature   

For the synthesis and spectroscopic data, see: Turner (1983 ▸). For related compounds, see: El-Hiti *et al.* (2015*a* ▸,*b* ▸); de Candia *et al.* (2013 ▸); Smith *et al.* (2013 ▸, 2012 ▸); Abdel-Megeed *et al.* (2012 ▸); Joule & Mills (2000 ▸). For the crystal structures of related compounds, see: El-Hiti *et al.* (2014 ▸); Seidler *et al.* (2011 ▸); Koch *et al.* (2008 ▸).
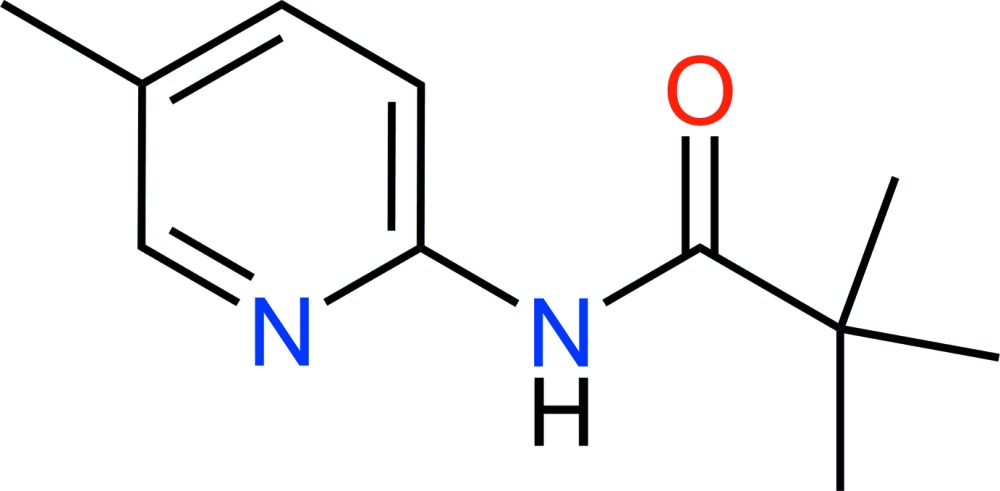


## Experimental   

### Crystal data  

C_11_H_16_N_2_O
*M* *_r_* = 192.26
Monoclinic, 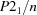

*a* = 11.1969 (2) Å
*b* = 8.6439 (2) Å
*c* = 23.8844 (5) Å
β = 94.549 (2)°
*V* = 2304.37 (8) Å^3^
*Z* = 8
Cu *K*α radiation
μ = 0.57 mm^−1^
*T* = 296 K
0.47 × 0.33 × 0.11 mm

### Data collection  

Agilent SuperNova (Dual, Cu at zero, Atlas) diffractometer
Absorption correction: gaussian (*CrysAlis PRO*; Agilent, 2014 ▸) *T*_min_ = 0.925, *T*_max_ = 0.975
8391 measured reflections
4503 independent reflections
3684 reflections with *I* > 2σ(*I*)
*R*_int_ = 0.021

### Refinement  

*R*[*F* ^2^ > 2σ(*F* ^2^)] = 0.047
*wR*(*F* ^2^) = 0.148
*S* = 1.04
4503 reflections
262 parameters
H-atom parameters constrained
Δρ_max_ = 0.27 e Å^−3^
Δρ_min_ = −0.21 e Å^−3^

### 

Data collection: *CrysAlis PRO* (Agilent, 2014 ▸); cell refinement: *CrysAlis PRO*; data reduction: *CrysAlis PRO*; program(s) used to solve structure: *SHELXS2013* (Sheldrick, 2015 ▸); program(s) used to refine structure: *SHELXL2013* (Sheldrick, 2015 ▸); molecular graphics: *ORTEP-3 for Windows* (Farrugia, 2012 ▸); software used to prepare material for publication: *WinGX* (Farrugia, 2012 ▸).

## Supplementary Material

Crystal structure: contains datablock(s) I, New_Global_Publ_Block. DOI: 10.1107/S2056989015009378/hb7430sup1.cif

Structure factors: contains datablock(s) I. DOI: 10.1107/S2056989015009378/hb7430Isup2.hkl

Supporting information file. DOI: 10.1107/S2056989015009378/hb7430Isup3.cml

**Fig. 1.**
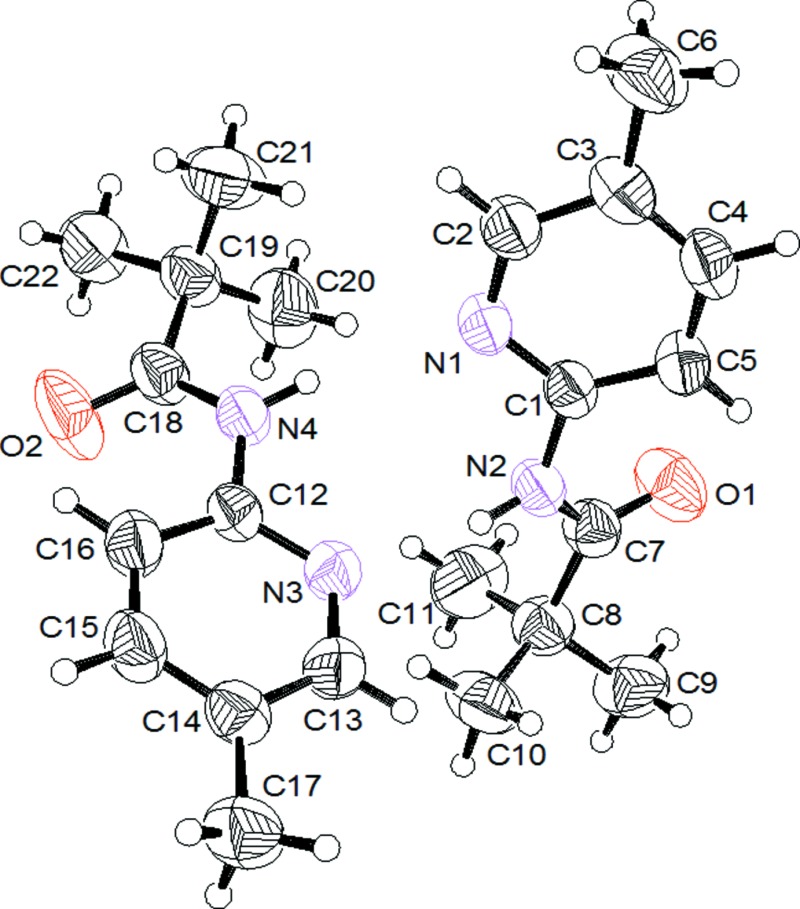
The asymmetric unit of C11H16N2O with 50% probability displacement ellipsoids for nonhydrogen atoms.

11 16 2 . DOI: 10.1107/S2056989015009378/hb7430fig1.tif
The asymmetric unit of C_11_H_16_N_2_O with 50% probability displacement ellipsoids for nonhydrogen atoms.

**Fig. 2.**
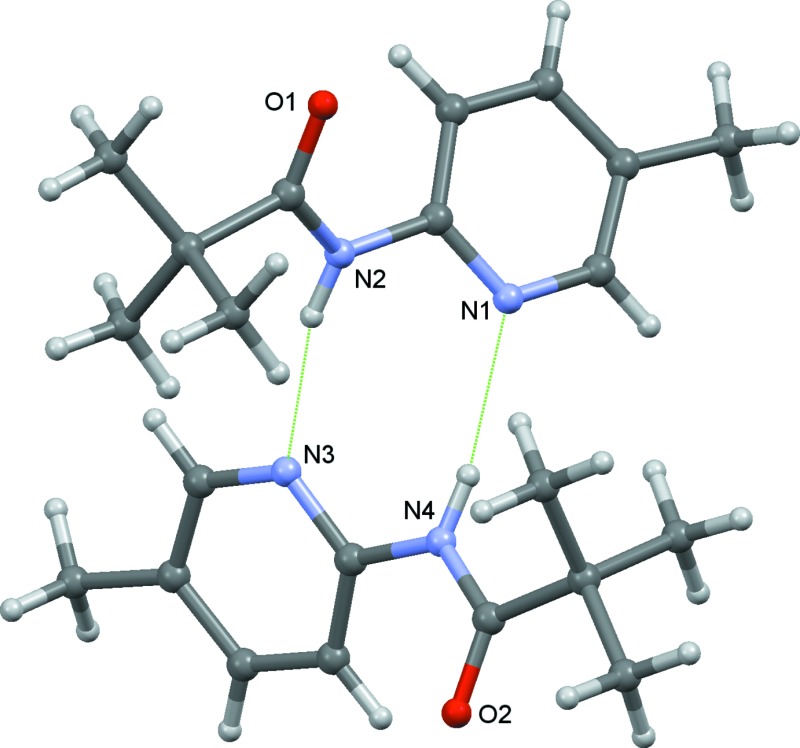
The asymmetric unit showing N—H···N interactions as dotted lines.

. DOI: 10.1107/S2056989015009378/hb7430fig2.tif
The asymmetric unit showing N—H⋯N inter­actions as dotted lines.

CCDC reference: 1401551

Additional supporting information:  crystallographic information; 3D view; checkCIF report

## Figures and Tables

**Table 1:** Hydrogen-bond geometry (, )

*D*H*A*	*D*H	H*A*	*D* *A*	*D*H*A*
N2H2*A*N3	0.86	2.31	3.1192(16)	156
N4H4*A*N1	0.86	2.25	3.0837(16)	163
C4H4O2^i^	0.93	2.43	3.3262(19)	160

Symmetry code: (i) 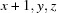.

## supplementary crystallographic information

### S1. Introduction

Substituted pyridines are important compounds (Joule & Mills, 2000) and show a range of biological activities (de Candia *et al.*, 2013; Abdel-Megeed *et al.*, 2012). The pyridine ring system has been modified using various efficient and simple procedures that include the use of lithium reagents as inter­mediates (El-Hiti *et al.*, 2015; Smith *et al.*, 2013, Smith *et al.*, 2012; Turner, 1983). The X-ray crystal structures of related compounds have been reported (El-Hiti *et al.*, 2015; El-Hiti *et al.*, 2014; Seidler *et al.*, 2011; Koch *et al.*, 2008).

### S2.1. Synthesis and crystallization

2,2-Di­methyl-*N*-(5-methyl­pyridin-2-yl)propanamide was obtained in 83% yield from reaction of 2-amino-5-methyl­pyridine with tri­methyl­acetyl chloride in the presence of tri­ethyl­amine in di­chloro­methane at 0 °C for 15 minutes and then at room temperature for 2 h (Turner, 1983). The crude product was purified by column chromatography (silica gel; di­chloro­methane) followed by crystallization from hexane to give colourless crystals of the title compound. The NMR spectral data and elemental analyses for the title compound were identical with those previously reported (Turner, 1983).

### S2.2. Refinement

H atoms were positioned geometrically and refined using a riding model with *U*_iso_(H) constrained to be 1.2 times *U*_eq_ for the atom it is bonded to except for methyl groups where it was 1.5 times with free rotation about the C—C bond.

### S3. Results and discussion

The asymmetric unit (Figure 1) consists of two independent molecules of C_11_H_16_N_2_O. The amide group and pyridine ring within the molecule are not co-planar as indicated by the torsion angles [N1—C1—N2—C7 = 142.20 (14), N3—C12—N4—C18 = 148.58 (15)]. The two independent molecules are linked by a pair of N—H···N hydrogen bonds (Table 1, Figure 2) to form an R_2_^2^(8) ring.

### Crystal data

**Table:**

C_11_H_16_N_2_O	*F*(000) = 832
*M**_r_* = 192.26	*D*_x_ = 1.108 Mg m^−^^3^
Monoclinic, *P*2_1_/*n*	Cu *K*α radiation, λ = 1.54184 Å
*a* = 11.1969 (2) Å	Cell parameters from 3890 reflections
*b* = 8.6439 (2) Å	θ = 3.9–73.6°
*c* = 23.8844 (5) Å	µ = 0.57 mm^−^^1^
β = 94.549 (2)°	*T* = 296 K
*V* = 2304.37 (8) Å^3^	Plate, colourless
*Z* = 8	0.47 × 0.33 × 0.11 mm

### Data collection

**Table:**

Agilent SuperNova (Dual, Cu at zero, Atlas) diffractometer	4503 independent reflections
Radiation source: sealed X-ray tube, SuperNova (Cu) X-ray Source	3684 reflections with *I* > 2σ(*I*)
Mirror monochromator	*R*_int_ = 0.021
ω scans	θ_max_ = 74.0°, θ_min_ = 3.7°
Absorption correction: gaussian (*CrysAlis PRO*; Agilent, 2014)	*h* = −13→13
*T*_min_ = 0.925, *T*_max_ = 0.975	*k* = −9→10
8391 measured reflections	*l* = −29→24

### Refinement

**Table:**

Refinement on *F*^2^	Hydrogen site location: inferred from neighbouring sites
Least-squares matrix: full	H-atom parameters constrained
*R*[*F*^2^ > 2σ(*F*^2^)] = 0.047	*w* = 1/[σ^2^(*F*_o_^2^) + (0.081*P*)^2^ + 0.208*P*] where *P* = (*F*_o_^2^ + 2*F*_c_^2^)/3
*wR*(*F*^2^) = 0.148	(Δ/σ)_max_ < 0.001
*S* = 1.04	Δρ_max_ = 0.27 e Å^−^^3^
4503 reflections	Δρ_min_ = −0.21 e Å^−^^3^
262 parameters	Extinction correction: *SHELXL2013* (Sheldrick, 2015), Fc^*^=kFc[1+0.001xFc^2^λ^3^/sin(2θ)]^-1/4^
0 restraints	Extinction coefficient: 0.0075 (6)

### Special details

**Table:**

Experimental. Absorption correction: CrysAlisPro, Agilent Technologies, Version 1.171.37.33 (release 27-03-2014 CrysAlis171 .NET) (compiled Mar 27 2014,17:12:48) Numerical absorption correction based on gaussian integration over a multifaceted crystal model Empirical absorption correction using spherical harmonics, implemented in SCALE3 ABSPACK scaling algorithm.
Geometry. All e.s.d.'s (except the e.s.d. in the dihedral angle between two l.s. planes) are estimated using the full covariance matrix. The cell e.s.d.'s are taken into account individually in the estimation of e.s.d.'s in distances, angles and torsion angles; correlations between e.s.d.'s in cell parameters are only used when they are defined by crystal symmetry. An approximate (isotropic) treatment of cell e.s.d.'s is used for estimating e.s.d.'s involving l.s. planes.

### Fractional atomic coordinates and isotropic or equivalent isotropic displacement parameters (Å^2^)

**Table:**

	*x*	*y*	*z*	*U*_iso_*/*U*_eq_	
C1	0.20122 (11)	0.54209 (16)	0.13526 (5)	0.0525 (3)	
C2	0.19343 (14)	0.77138 (18)	0.08875 (7)	0.0650 (4)	
H2	0.1480	0.8535	0.0736	0.097*	
C3	0.31560 (14)	0.77581 (19)	0.08401 (7)	0.0670 (4)	
C4	0.38057 (13)	0.6528 (2)	0.10739 (7)	0.0701 (4)	
H4	0.4632	0.6499	0.1055	0.105*	
C5	0.32450 (12)	0.5351 (2)	0.13327 (7)	0.0654 (4)	
H5	0.3681	0.4524	0.1492	0.098*	
C6	0.3730 (2)	0.9087 (3)	0.05543 (12)	0.1036 (7)	
H6A	0.4566	0.9132	0.0677	0.155*	
H6B	0.3349	1.0037	0.0649	0.155*	
H6C	0.3639	0.8939	0.0155	0.155*	
C7	0.15945 (14)	0.27433 (18)	0.16115 (6)	0.0618 (4)	
C8	0.07563 (15)	0.16852 (19)	0.19135 (7)	0.0689 (4)	
C9	0.1450 (2)	0.0226 (2)	0.21008 (9)	0.0942 (6)	
H9A	0.1777	−0.0243	0.1782	0.141*	
H9B	0.0920	−0.0490	0.2263	0.141*	
H9C	0.2090	0.0496	0.2375	0.141*	
C10	0.0273 (2)	0.2443 (2)	0.24272 (9)	0.0918 (6)	
H10A	−0.0163	0.1692	0.2626	0.138*	
H10B	−0.0250	0.3280	0.2309	0.138*	
H10C	0.0929	0.2832	0.2670	0.138*	
C11	−0.0263 (2)	0.1254 (3)	0.14762 (12)	0.1106 (8)	
H11A	0.0066	0.0838	0.1149	0.166*	
H11B	−0.0726	0.2160	0.1374	0.166*	
H11C	−0.0768	0.0494	0.1631	0.166*	
C12	−0.12246 (11)	0.71207 (16)	0.19991 (6)	0.0520 (3)	
C13	−0.02137 (14)	0.65237 (19)	0.28397 (6)	0.0650 (4)	
H13	0.0409	0.5995	0.3037	0.098*	
C14	−0.09434 (14)	0.74294 (19)	0.31429 (6)	0.0634 (4)	
C15	−0.18413 (15)	0.8222 (2)	0.28376 (7)	0.0710 (4)	
H15	−0.2350	0.8861	0.3023	0.106*	
C16	−0.19970 (14)	0.8082 (2)	0.22610 (7)	0.0660 (4)	
H16	−0.2603	0.8618	0.2054	0.099*	
C17	−0.0774 (2)	0.7526 (3)	0.37741 (7)	0.0909 (6)	
H17A	−0.0997	0.8538	0.3894	0.136*	
H17B	0.0051	0.7335	0.3895	0.136*	
H17C	−0.1268	0.6765	0.3936	0.136*	
C18	−0.22818 (13)	0.6925 (2)	0.10586 (6)	0.0671 (4)	
C19	−0.21118 (14)	0.6626 (2)	0.04398 (6)	0.0714 (4)	
C20	−0.1518 (2)	0.5048 (3)	0.03776 (9)	0.1036 (7)	
H20A	−0.0736	0.5054	0.0575	0.155*	
H20B	−0.1445	0.4838	−0.0013	0.155*	
H20C	−0.1999	0.4260	0.0532	0.155*	
C21	−0.1325 (2)	0.7892 (3)	0.02172 (9)	0.1074 (8)	
H21A	−0.1659	0.8887	0.0293	0.161*	
H21B	−0.1291	0.7767	−0.0181	0.161*	
H21C	−0.0532	0.7819	0.0400	0.161*	
C22	−0.33408 (17)	0.6641 (3)	0.01113 (8)	0.0955 (7)	
H22A	−0.3810	0.5790	0.0230	0.143*	
H22B	−0.3242	0.6546	−0.0283	0.143*	
H22C	−0.3742	0.7596	0.0180	0.143*	
N1	0.13561 (10)	0.65829 (14)	0.11338 (5)	0.0578 (3)	
N2	0.13689 (10)	0.42840 (14)	0.16285 (5)	0.0577 (3)	
H2A	0.0795	0.4589	0.1821	0.086*	
N3	−0.03374 (10)	0.63507 (14)	0.22795 (5)	0.0589 (3)	
N4	−0.12739 (10)	0.69100 (15)	0.14133 (5)	0.0585 (3)	
H4A	−0.0606	0.6758	0.1267	0.088*	
O1	0.23876 (14)	0.22212 (15)	0.13479 (7)	0.0942 (4)	
O2	−0.32545 (10)	0.7145 (3)	0.12290 (6)	0.1234 (7)	

### Atomic displacement parameters (Å^2^)

**Table:**

	*U*^11^	*U*^22^	*U*^33^	*U*^12^	*U*^13^	*U*^23^
C1	0.0471 (6)	0.0604 (7)	0.0506 (6)	−0.0005 (5)	0.0069 (5)	−0.0061 (5)
C2	0.0609 (8)	0.0606 (8)	0.0750 (9)	0.0020 (6)	0.0152 (7)	0.0019 (7)
C3	0.0619 (8)	0.0670 (9)	0.0739 (9)	−0.0103 (7)	0.0180 (7)	−0.0082 (7)
C4	0.0458 (7)	0.0872 (11)	0.0783 (10)	−0.0058 (7)	0.0111 (6)	−0.0054 (8)
C5	0.0476 (7)	0.0792 (10)	0.0695 (9)	0.0046 (7)	0.0061 (6)	0.0024 (7)
C6	0.0929 (14)	0.0845 (13)	0.139 (2)	−0.0182 (11)	0.0419 (13)	0.0105 (12)
C7	0.0633 (8)	0.0634 (8)	0.0595 (8)	0.0033 (7)	0.0093 (6)	−0.0058 (6)
C8	0.0743 (10)	0.0600 (8)	0.0730 (9)	−0.0017 (7)	0.0096 (8)	0.0039 (7)
C9	0.1260 (17)	0.0716 (11)	0.0864 (12)	0.0159 (11)	0.0172 (12)	0.0096 (9)
C10	0.1089 (15)	0.0753 (11)	0.0977 (13)	0.0020 (10)	0.0486 (12)	0.0135 (10)
C11	0.0982 (15)	0.1086 (17)	0.1210 (18)	−0.0325 (13)	−0.0168 (14)	0.0207 (14)
C12	0.0441 (6)	0.0584 (7)	0.0541 (7)	−0.0037 (5)	0.0082 (5)	−0.0028 (5)
C13	0.0649 (8)	0.0748 (9)	0.0553 (8)	0.0084 (7)	0.0041 (6)	0.0003 (7)
C14	0.0628 (8)	0.0718 (9)	0.0566 (8)	−0.0045 (7)	0.0101 (6)	−0.0074 (6)
C15	0.0643 (9)	0.0830 (10)	0.0672 (9)	0.0103 (8)	0.0145 (7)	−0.0160 (8)
C16	0.0574 (8)	0.0769 (9)	0.0643 (8)	0.0125 (7)	0.0078 (6)	−0.0056 (7)
C17	0.0947 (13)	0.1195 (16)	0.0590 (9)	0.0039 (12)	0.0092 (9)	−0.0124 (10)
C18	0.0479 (7)	0.0957 (11)	0.0581 (8)	−0.0060 (7)	0.0062 (6)	−0.0059 (7)
C19	0.0574 (8)	0.1030 (12)	0.0540 (8)	−0.0056 (8)	0.0058 (6)	−0.0037 (8)
C20	0.1021 (15)	0.1323 (18)	0.0765 (12)	0.0172 (14)	0.0078 (10)	−0.0312 (12)
C21	0.0894 (13)	0.161 (2)	0.0718 (11)	−0.0318 (14)	0.0046 (10)	0.0262 (13)
C22	0.0681 (10)	0.153 (2)	0.0636 (10)	−0.0080 (12)	−0.0058 (8)	−0.0082 (11)
N1	0.0483 (6)	0.0612 (6)	0.0649 (7)	0.0024 (5)	0.0111 (5)	−0.0014 (5)
N2	0.0517 (6)	0.0616 (7)	0.0612 (6)	0.0020 (5)	0.0141 (5)	0.0003 (5)
N3	0.0564 (6)	0.0654 (7)	0.0555 (6)	0.0082 (5)	0.0078 (5)	−0.0030 (5)
N4	0.0464 (6)	0.0772 (8)	0.0526 (6)	0.0019 (5)	0.0081 (5)	−0.0045 (5)
O1	0.1027 (10)	0.0738 (8)	0.1127 (10)	0.0100 (7)	0.0496 (8)	−0.0114 (7)
O2	0.0451 (6)	0.250 (2)	0.0754 (8)	−0.0005 (9)	0.0067 (5)	−0.0370 (11)

### Geometric parameters (Å, º)

**Table:**

C1—N1	1.3269 (18)	C12—C16	1.384 (2)
C1—C5	1.3862 (19)	C12—N4	1.4077 (17)
C1—N2	1.4122 (17)	C13—N3	1.3427 (19)
C2—N1	1.3346 (19)	C13—C14	1.378 (2)
C2—C3	1.382 (2)	C13—H13	0.9300
C2—H2	0.9300	C14—C15	1.377 (2)
C3—C4	1.381 (2)	C14—C17	1.507 (2)
C3—C6	1.506 (2)	C15—C16	1.380 (2)
C4—C5	1.368 (2)	C15—H15	0.9300
C4—H4	0.9300	C16—H16	0.9300
C5—H5	0.9300	C17—H17A	0.9600
C6—H6A	0.9600	C17—H17B	0.9600
C6—H6B	0.9600	C17—H17C	0.9600
C6—H6C	0.9600	C18—O2	1.208 (2)
C7—O1	1.2155 (19)	C18—N4	1.3565 (19)
C7—N2	1.3567 (19)	C18—C19	1.527 (2)
C7—C8	1.531 (2)	C19—C21	1.526 (3)
C8—C10	1.527 (3)	C19—C22	1.529 (2)
C8—C9	1.529 (3)	C19—C20	1.531 (3)
C8—C11	1.531 (3)	C20—H20A	0.9600
C9—H9A	0.9600	C20—H20B	0.9600
C9—H9B	0.9600	C20—H20C	0.9600
C9—H9C	0.9600	C21—H21A	0.9600
C10—H10A	0.9600	C21—H21B	0.9600
C10—H10B	0.9600	C21—H21C	0.9600
C10—H10C	0.9600	C22—H22A	0.9600
C11—H11A	0.9600	C22—H22B	0.9600
C11—H11B	0.9600	C22—H22C	0.9600
C11—H11C	0.9600	N2—H2A	0.8600
C12—N3	1.3314 (18)	N4—H4A	0.8600
			
N1—C1—C5	122.76 (13)	N3—C13—H13	117.7
N1—C1—N2	115.06 (11)	C14—C13—H13	117.7
C5—C1—N2	122.13 (13)	C15—C14—C13	116.30 (14)
N1—C2—C3	125.05 (15)	C15—C14—C17	122.08 (15)
N1—C2—H2	117.5	C13—C14—C17	121.62 (16)
C3—C2—H2	117.5	C14—C15—C16	120.94 (14)
C4—C3—C2	116.01 (14)	C14—C15—H15	119.5
C4—C3—C6	122.73 (16)	C16—C15—H15	119.5
C2—C3—C6	121.25 (17)	C15—C16—C12	118.01 (14)
C5—C4—C3	120.60 (14)	C15—C16—H16	121.0
C5—C4—H4	119.7	C12—C16—H16	121.0
C3—C4—H4	119.7	C14—C17—H17A	109.5
C4—C5—C1	118.49 (15)	C14—C17—H17B	109.5
C4—C5—H5	120.8	H17A—C17—H17B	109.5
C1—C5—H5	120.8	C14—C17—H17C	109.5
C3—C6—H6A	109.5	H17A—C17—H17C	109.5
C3—C6—H6B	109.5	H17B—C17—H17C	109.5
H6A—C6—H6B	109.5	O2—C18—N4	121.25 (14)
C3—C6—H6C	109.5	O2—C18—C19	122.54 (14)
H6A—C6—H6C	109.5	N4—C18—C19	116.20 (13)
H6B—C6—H6C	109.5	C21—C19—C18	109.61 (16)
O1—C7—N2	121.70 (15)	C21—C19—C22	109.63 (17)
O1—C7—C8	121.50 (15)	C18—C19—C22	108.60 (14)
N2—C7—C8	116.72 (13)	C21—C19—C20	109.78 (18)
C10—C8—C9	108.82 (16)	C18—C19—C20	109.45 (16)
C10—C8—C11	111.11 (19)	C22—C19—C20	109.75 (18)
C9—C8—C11	109.45 (17)	C19—C20—H20A	109.5
C10—C8—C7	113.06 (14)	C19—C20—H20B	109.5
C9—C8—C7	108.31 (15)	H20A—C20—H20B	109.5
C11—C8—C7	106.00 (15)	C19—C20—H20C	109.5
C8—C9—H9A	109.5	H20A—C20—H20C	109.5
C8—C9—H9B	109.5	H20B—C20—H20C	109.5
H9A—C9—H9B	109.5	C19—C21—H21A	109.5
C8—C9—H9C	109.5	C19—C21—H21B	109.5
H9A—C9—H9C	109.5	H21A—C21—H21B	109.5
H9B—C9—H9C	109.5	C19—C21—H21C	109.5
C8—C10—H10A	109.5	H21A—C21—H21C	109.5
C8—C10—H10B	109.5	H21B—C21—H21C	109.5
H10A—C10—H10B	109.5	C19—C22—H22A	109.5
C8—C10—H10C	109.5	C19—C22—H22B	109.5
H10A—C10—H10C	109.5	H22A—C22—H22B	109.5
H10B—C10—H10C	109.5	C19—C22—H22C	109.5
C8—C11—H11A	109.5	H22A—C22—H22C	109.5
C8—C11—H11B	109.5	H22B—C22—H22C	109.5
H11A—C11—H11B	109.5	C1—N1—C2	117.08 (12)
C8—C11—H11C	109.5	C7—N2—C1	124.47 (12)
H11A—C11—H11C	109.5	C7—N2—H2A	117.8
H11B—C11—H11C	109.5	C1—N2—H2A	117.8
N3—C12—C16	122.75 (13)	C12—N3—C13	117.35 (12)
N3—C12—N4	113.83 (11)	C18—N4—C12	125.79 (12)
C16—C12—N4	123.38 (13)	C18—N4—H4A	117.1
N3—C13—C14	124.64 (14)	C12—N4—H4A	117.1
			
N1—C2—C3—C4	−0.6 (3)	O2—C18—C19—C21	119.0 (2)
N1—C2—C3—C6	−179.97 (18)	N4—C18—C19—C21	−61.9 (2)
C2—C3—C4—C5	0.2 (2)	O2—C18—C19—C22	−0.8 (3)
C6—C3—C4—C5	179.56 (18)	N4—C18—C19—C22	178.35 (18)
C3—C4—C5—C1	0.3 (2)	O2—C18—C19—C20	−120.6 (2)
N1—C1—C5—C4	−0.3 (2)	N4—C18—C19—C20	58.5 (2)
N2—C1—C5—C4	−177.72 (14)	C5—C1—N1—C2	0.0 (2)
O1—C7—C8—C10	151.67 (19)	N2—C1—N1—C2	177.54 (12)
N2—C7—C8—C10	−31.6 (2)	C3—C2—N1—C1	0.5 (2)
O1—C7—C8—C9	31.0 (2)	O1—C7—N2—C1	−0.2 (2)
N2—C7—C8—C9	−152.30 (15)	C8—C7—N2—C1	−176.94 (13)
O1—C7—C8—C11	−86.4 (2)	N1—C1—N2—C7	142.20 (14)
N2—C7—C8—C11	90.33 (19)	C5—C1—N2—C7	−40.2 (2)
N3—C13—C14—C15	−1.3 (3)	C16—C12—N3—C13	0.6 (2)
N3—C13—C14—C17	177.95 (17)	N4—C12—N3—C13	178.41 (13)
C13—C14—C15—C16	0.9 (3)	C14—C13—N3—C12	0.5 (2)
C17—C14—C15—C16	−178.27 (18)	O2—C18—N4—C12	0.7 (3)
C14—C15—C16—C12	0.1 (3)	C19—C18—N4—C12	−178.44 (15)
N3—C12—C16—C15	−0.9 (2)	N3—C12—N4—C18	148.58 (15)
N4—C12—C16—C15	−178.46 (15)	C16—C12—N4—C18	−33.7 (2)

### Hydrogen-bond geometry (Å, º)

**Table:**

*D*—H···*A*	*D*—H	H···*A*	*D*···*A*	*D*—H···*A*
N2—H2*A*···N3	0.86	2.31	3.1192 (16)	156
N4—H4*A*···N1	0.86	2.25	3.0837 (16)	163
C4—H4···O2^i^	0.93	2.43	3.3262 (19)	160

Symmetry code: (i) *x*+1, *y*, *z*.
