# Supplementary figures and images for: Crystal structure of 2,2-dimethyl-N-(5-methyl­pyridin-2-yl)propanamide
Source: Acta Crystallogr E Crystallogr Commun. 2015 May 23;71(Pt 6):o419–20. doi: 10.1107/S2056989015009378 (PMC4459364; doi:10.1107/S2056989015009378)

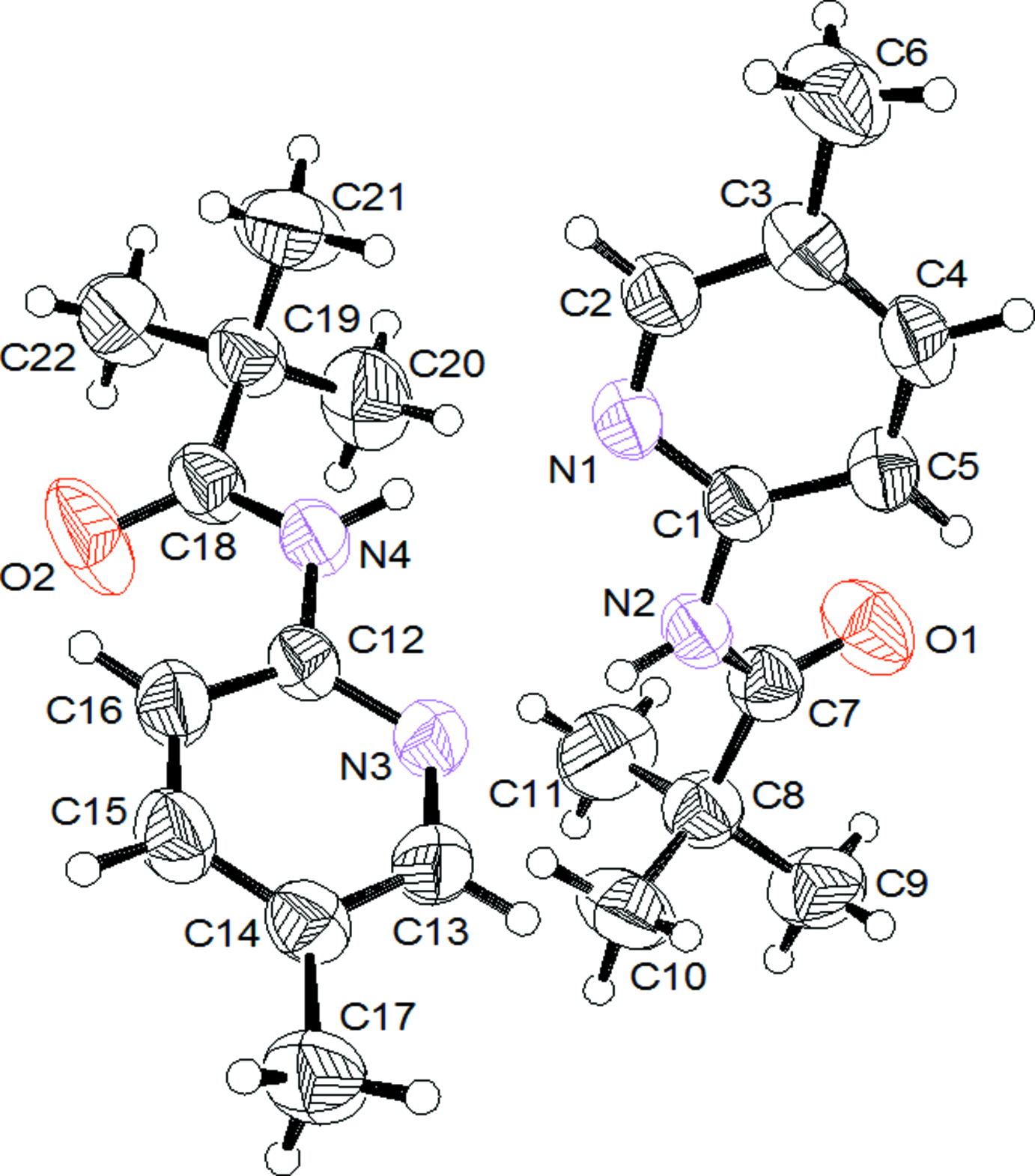

Supplement: Supplementary file 4 [file e-71-0o419-fig1.tif]

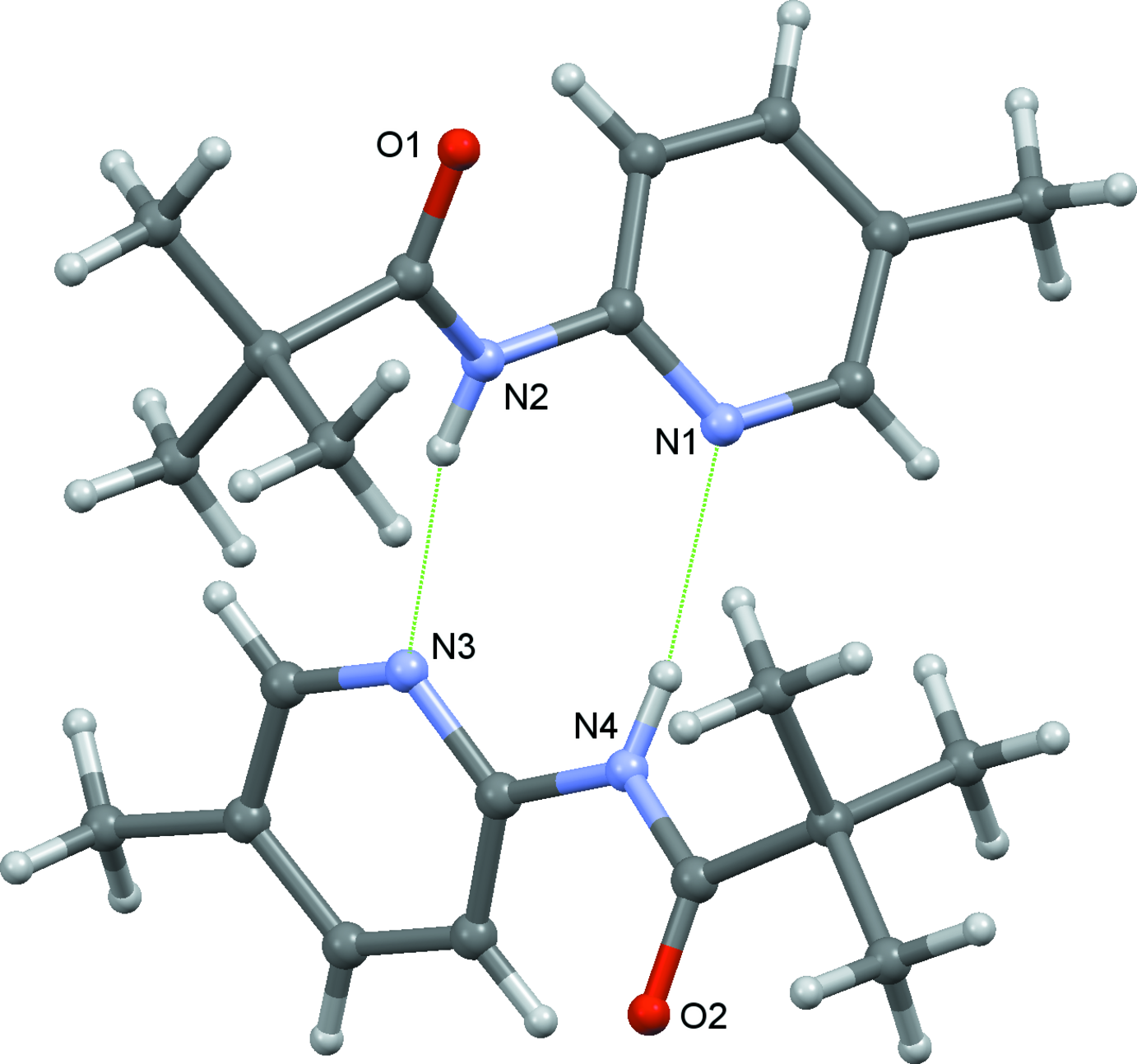

Supplement: Supplementary file 5 [file e-71-0o419-fig2.tif]
